# Differential fatty acids utilization across life stages in a Vespa species

**DOI:** 10.1007/s00360-024-01589-7

**Published:** 2024-10-10

**Authors:** Sofia Bouchebti, Eran Levin

**Affiliations:** 1 School of Zoology, George S. Wise Faculty of Life Sciences, Tel Aviv University, Tel Aviv, 6997801 Israel; 2 Present address: Mitrani Department of Desert Ecology, Blaustein Institutes for Desert Research, Ben-Gurion University of the Negev, Midreshet Ben Gurion, 8499000 Israel

**Keywords:** *Vespa orientalis*, SFA, MUFA, PUFA, Stable carbon isotope

## Abstract

Dietary fatty acids (FAs) are essential macronutrients affecting animal fitness, growth, and development. While the degree of saturation of FAs usually determines the level of absorption and allocation within the body, the utilization of dietary FAs across the life stages of individuals remains unknown. We used three different 13 C labeled FAs, with a different saturation level (linoleic acid (18:2), oleic acid (18:1), and palmitic acid (16:0)), to investigate the absorption and allocation of dietary FAs across the life stages of the Oriental hornet. Our results show that only larvae utilized all tested FAs as metabolic fuel, with palmitic acid being oxidized at the highest rate. Oleic and palmitic acids were predominantly incorporated into larval tissues, while oleic acid dominated pupal tissues. In contrast, linoleic and oleic acids were predominantly incorporated into adult tissues. These findings highlight a life stage-dependent shift in certain FAs utilization, with palmitic acid mostly utilized in early stages and linoleic acid in adulthood, while oleic acid remained consistently utilized across all life stages. This study emphasizes the importance of considering FA saturation and life stage dynamics in understanding FA utilization patterns.

## Introduction

Fatty acids (FAs) constitute essential macronutrients, they are a vital fuel source for many animals and have different critical biological functions (De Carvalho and Caramujo 2018). FAs can be either synthesized de novo or acquired from dietary sources (De Carvalho and Caramujo 2018). Imbalances in the intake of dietary FAs, either in excess or deprivation, can detrimentally affect animal fitness, impair growth, and hinder development in both vertebrates and invertebrates (Canavoso et al. 2001; Innis 1991; Ruedenauer et al. 2020). Consequently, it was suggested that many animals possess the ability to detect FAs within their food and regulate their consumption accordingly (Kraus et al. 2019; Ruedenauer et al. 2020, 2023; Stabler et al. 2021; Vaudo et al. 2016; Vaudo et al. 2016).

FAs are categorized into three primary groups based on their double bond count: saturated FAs (SFAs), monounsaturated FAs (MUFAs), and polyunsaturated FAs (PUFAs) (De Carvalho and Caramujo 2018). The degree of saturation of FAs impacts the fluidity, flexibility, and selective permeability of cellular membranes (De Carvalho and Caramujo 2018), while also determining their absorption and utilization within the body. A recent study showed that palmitic acid, a common SFA, was barely absorbed and oxidized in gecko and shrews when mixed in their diet but considerably egested compared to both linoleic acid (PUFA) and oleic acid (MUFA), demonstrating distinct ingestion patterns according to FA saturation levels (Dubiner et al. 2023). Aside from saturation levels, the chemical environment in which FAs are consumed also contributes to the degree of oxidation and integration into bodily tissues, albeit to a lesser degree (Seltzer et al. 2023).

While most research on the utilization of dietary FAs has focused on adult individuals, it is important to note that nutritional requirements change across different life stages, with FAs holding particular significance during juvenile phases (Canavoso et al. 2001; Innis 1991). As a result, the oxidation and allocation of dietary FAs may differ according to the individual’s life stage. In this study, we compared the oxidation and allocation of different FAs across different life stages of the Oriental hornet. Hornets are eusocial wasps feeding on nectar, fruits, insect prey, and carrion (Hunt 2007). While the digestive capacities of adult hornets are limited, larvae can efficiently metabolize complex nutrients such as protein and chitin (Bodner et al. 2022; Bouchebti et al. 2023; Hunt 2007). The larvae process nutrients collected by adults and subsequently shared among all colony members through a continuous nutrient exchange process (Bouchebti et al. 2022). Given that the presence of larvae is crucial for the digestion of certain nutrients, larvae may also play a role in facilitating the absorption of FAs in adult hornets. To investigate these hypotheses, we provided experimental hornet colonies with a diet enriched in three distinct isotopically labeled FAs: linoleic acid (PUFA), oleic acid (MUFA), and palmitic acid (SFA), all labeled by C_13_ on carbon no 1. We then measured the rates of oxidation and allocation of these FAs within both larvae and adults, with the latter being fed in the presence or absence of larvae.

## Materials and methods

### Experimental procedure

Four colonies of Oriental hornets were collected from areas around Tel Aviv University. The combs, containing eggs, larvae, and pupae, were excavated from the ground, and adult workers were collected from the excavated colonies using a sweeping net. Shortly after colonies’ collection, workers were placed in groups of three in experimental colonies confined in wooden boxes (10 × 14.4 × 12 cm). For the condition “with brood”, pieces of comb containing ten larvae (1st to 5th instars) were glued to the ceiling of the wooden boxes. Experimental colonies were supplied ad libitum with a test tube containing a 50% sucrose solution and an additional water tube. Raw minced beef meat (0.5 g) was provided daily. This quantity was chosen to ensure that some meat would always remain available until the next feeding. In the treatment groups, the minced meat was mixed with 1 mg of 13C_1_-labeled linoleic acid, 13C1-labeled oleic acid, or 13C1-labeled palmitic acid (Cambridge Isotope Laboratories, Tewksbury, MA, USA), while the minced meat in the control groups was provided without any fatty acid added. After seven days, the δ13C – i.e., the isotopic signature, which is the ratio of the two stable isotopes of carbon (13 C and 12 C) reported in per mille (‰) - in the respiration of the hornets (workers and 4th and 5th instars larvae) was analyzed, and individuals (workers, all instars’ larvae, and pupae) were killed by freezing. The pupae analyzed originated from larvae that had pupated during the experiment. The number of live larvae, pupae, and eggs laid by the workers were counted. The different tissues (larval body tissue, pupal body tissue, adult brain tissue, adult fat body tissue, and adult muscle tissue) were dissected and dried at 60 °C for three days.

For each treatment group—receiving meat enriched with 13C1-labeled linoleic acid, 13C1-labeled oleic acid, or 13C1-labeled palmitic acid—we performed eight replicates per condition (with and without brood). These replicates were distributed across the four colonies collected, with two replicates per colony. For the control group, which received meat without any added fatty acid, we performed four replicates per condition, with one replicate per colony.

To measure diet consumption, the meat for each treatment and condition was first weighed, placed in the wooden boxes, and weighed again 24 h later. The evaporation rate was assessed by placing and weighing each diet in control boxes, i.e., boxes without hornets. Four replicates were used for that purpose.

The experiments were conducted in a climate-controlled room (25 ± 2 °C, 75 ± 10% RH).

### Respiration δ13C analysis

To measure δ13CO_2_ in the respiration of the hornets, adults and larvae were placed individually in a 40 mL metabolic chamber, and their exhaled CO_2_ was recorded for 10 min. We used pull-mode respirometry as follows: room air was pulled at a constant rate (30 mL min − 1 STP) through an ascarite^®^ column (CO2 absorbent) into the metabolic chamber and then directly into a G2121-i cavity ring-down spectroscopy (CRDS) stable carbon isotope analyzer (Picarro, Santa Clara, CA)(Bodner et al. 2021). Total CO2 and δ13C were recorded every two seconds. All δ 13 C concentrations are expressed in δ13CVPDB.

### Tissues δ13C analysis

Samples of 1 mg of each dry tissue (larval body tissue, pupal body tissue, adult brain tissue, adult fat body tissue, and adult muscle tissue) were loaded into tin capsules. The δ13C (‰) values in the samples were measured and calculated using a Picarro G2121-i Cavity Ring-Down Spectroscopy δ13C stable isotope analyzer with an A0502 ambient CO_2_ interface, an A0201 Combustion Module, and an A0301 gas interface (CM-CRDS)(Bodner et al. 2021; Bouchebti et al. 2022). To confirm the analyzer’s calibration, we ran a secondary standard of verified δ13C value (sucrose) every ten samples. All 13 C concentrations are expressed in δ13CVPDB.

### Statistical analyses

The average δ13C values in the exhaled breath during the 10 min measurement were calculated for each individual. The average δ13C values in the exhaled breath and in different tissues were compared between groups for larvae and workers separately with linear mixed-effects models, followed by a post-hoc pairwise comparison (Tukey adjusted). The models were fitted by specifying the fixed factors (treatment: linoleic acid, oleic acid, palmitic acid, or control; and condition for the workers: with and without brood) and their interaction, and the individuals were nested in the colony as random factors. The δ13C values were log10-transformed (x - minimum value). The diet consumption and the fitness of the experimental colonies (i.e., number of larvae alive, pupae, and eggs) were also analyzed with linear mixed-effects models, followed by a post-hoc pairwise comparison (Tukey adjusted). The fixed factors included treatment, condition and their interaction for the diet consumption model, and treatment, type of brood (larvae, pupae, and eggs) and their interaction for the fitness model. For both models, the replicate was nested in colony as random factor, and the data were log10-transformed (x + 1). Statistical tests were run and graphics were generated on R 4.2.1 (R Core Team 2020).

## Results

### FAs as metabolic fuel

**Fig. 1:**
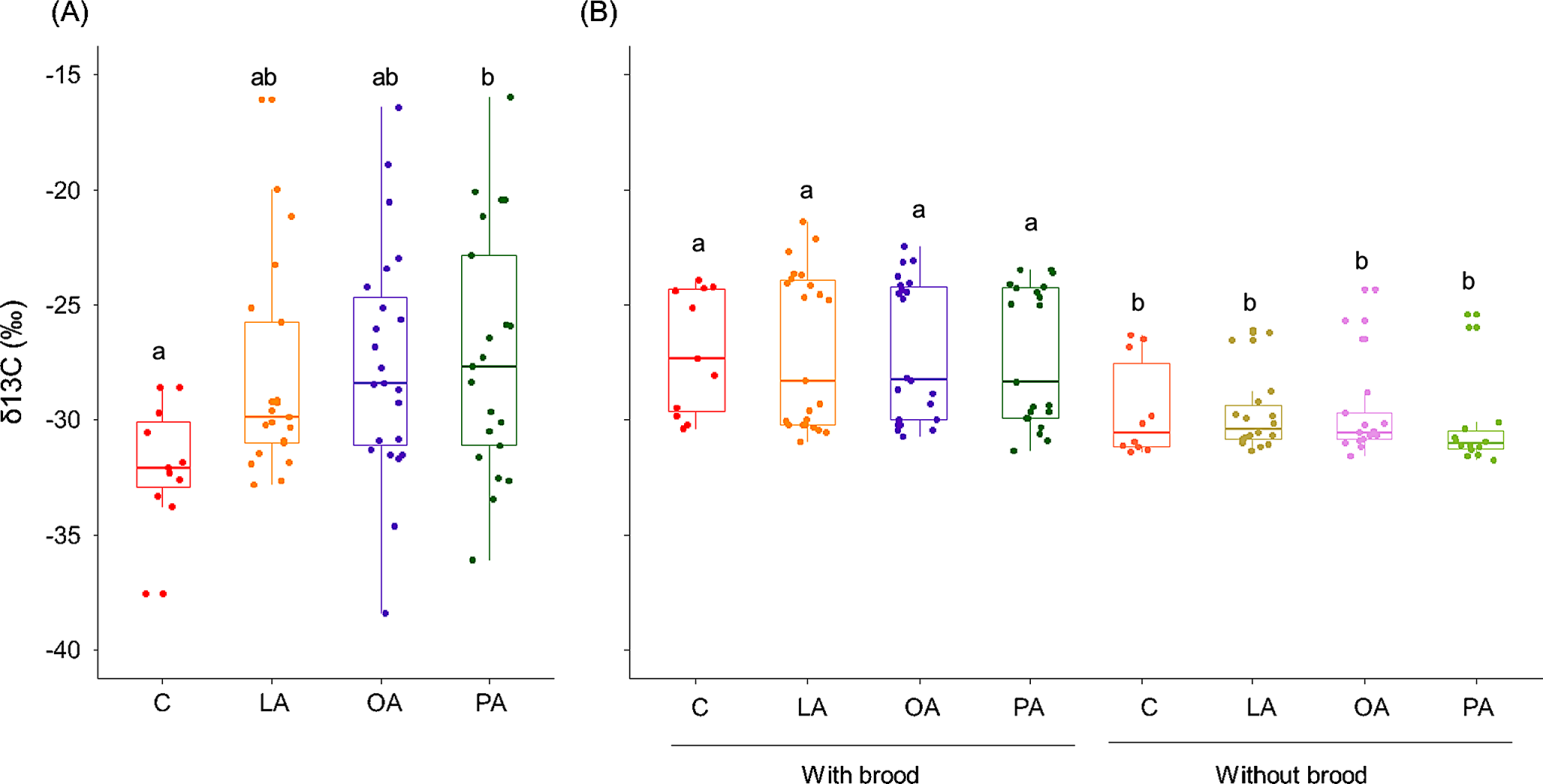
δ13C values (‰) in the CO_2_ from exhaled breath of (**A**) Larvae and (**B**) adult hornets fed with labeled FAs diet (LA: linoleic acid; OA: oleic acid; and PA: palmitic acid) and unlabeled diet (C: control). A higher delta indicates higher 13 C levels. The boxes represent the first and third quartiles and the median. The whiskers represent the maximum and minimum values. Groups that do not share the same letter are significantly different from each other (pairwise comparison with *P* < 0.05)

Larvae utilize (absorbed and oxidized) the three dietary FAs as metabolic fuel; however, they oxidized palmitic acid at a higher rate (Fig. 1A; F_3,45.35_ = 3.388, *P* = 0.026). Adult hornets, on the other hand, were not able to metabolize any of the FAs (Fig. 1B; Treatment: F_3, 111.70_ = 0.960, *P* = 0.414; Condition: F_1,111.69_ =83.246, *P* < 0.001; Treatment× Condition: F_3,108.15_ = 0.268, *P* = 0.848). The δ13C values in the CO_2_ from the exhaled breath of adult hornets were significantly higher in the presence of brood (Fig. 1B).

### FAs in body tissues

**Fig. 2:**
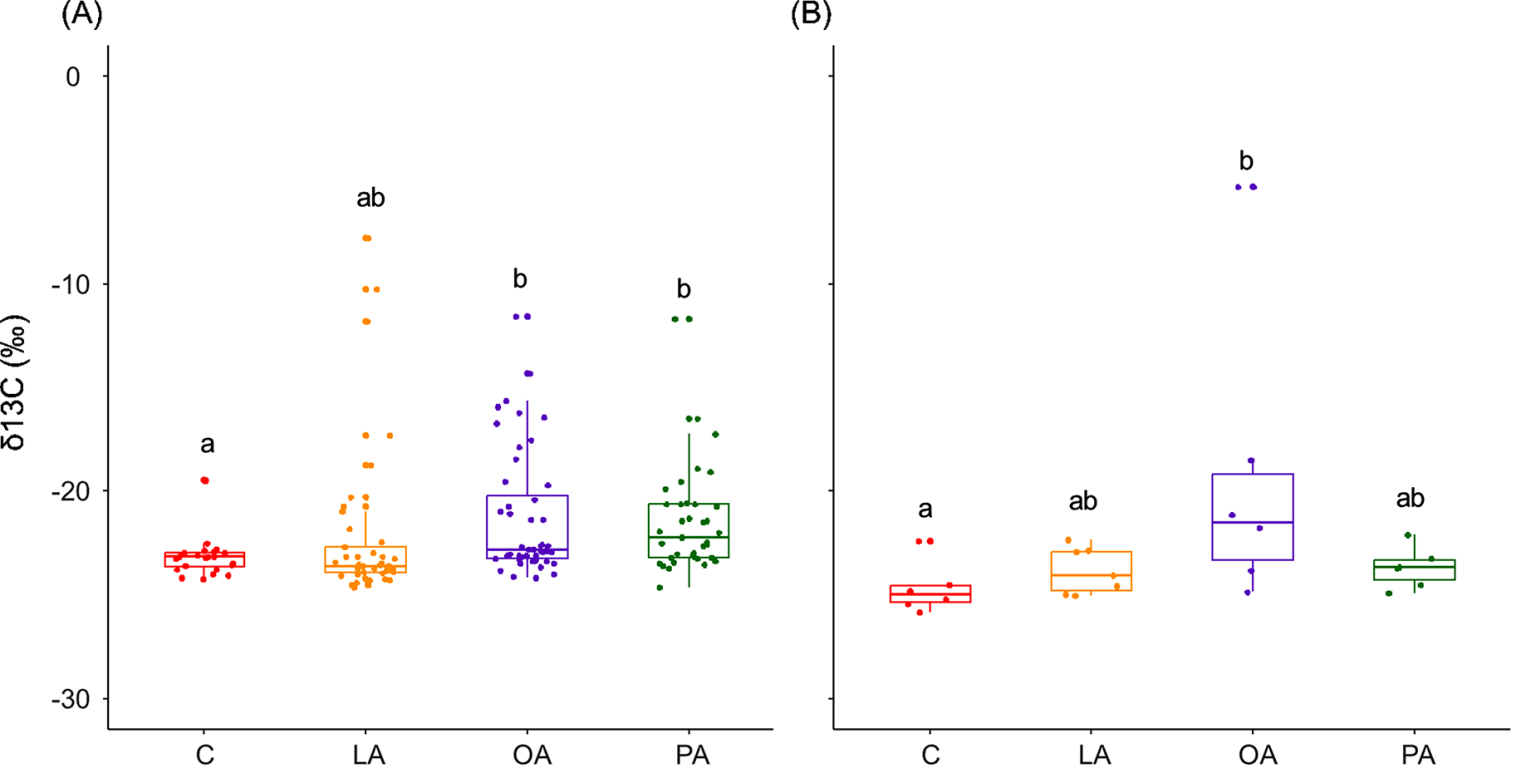
δ13C values (‰) in the body tissues of (**A**) larvae and (**B**) pupae fed with labeled FAs meat (LA: linoleic acid; OA: oleic acid; and PA: palmitic acid) and unlabeled meat (C: control). A higher delta indicates higher 13 C levels. The boxes represent the first and third quartiles and the median. The whiskers represent the maximum and minimum values. Groups that do not share the same letter are significantly different from each other (pairwise comparison with *P* < 0.05)

Larvae and pupae incorporated the three dietary FAs in their body tissues: while the δ13C were higher in the oleic and palmitic acids treatment in larval body tissues, in pupal body tissue, the oleic acid treatment had higher δ13C values (Fig. 2A &B; Larvae: F_3,142.46_= 3.909, *P* = 0.0102; Pupae: F_3,19.47_ =7.080, *P* = 0.002).

**Fig. 3:**
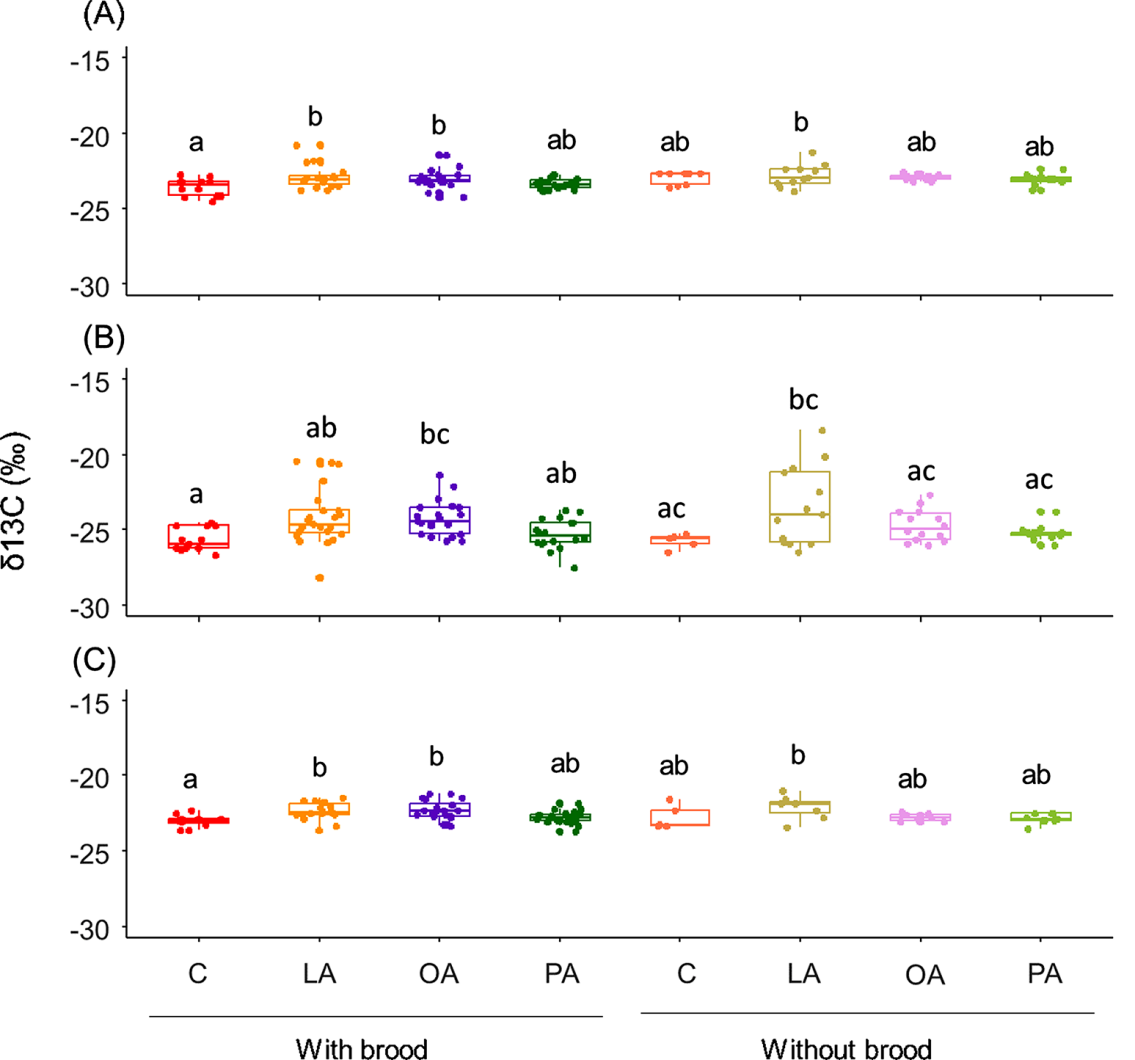
δ13C values (‰) in adult tissues, (**A**) brain, (**B**) fat body, and (**C**) muscles, fed with labeled FAs meat (LA: linoleic acid; OA: oleic acid; and PA: palmitic acid) and unlabeled meat (C: control). A higher delta indicates higher 13 C levels. The boxes represent the first and third quartiles and the median. The whiskers represent the maximum and minimum values. Groups that do not share the same letter are significantly different from each other (pairwise comparison with *P* < 0.05)

Adult hornets incorporated FAs in all of their tissues, however, unlike larvae, δ13C values were higher for the linoleic and oleic acids treatments in their brains (Fig. 3A; Treatment: F_3,94.36_ = 4.622, *P* = 0.005, Condition: F_1,104.45_ = 3.071, *P* = 0.083, Treatment× Condition: F _3,91.63_ = 0.971, *P* = 0.410), fat body (Fig. 3B; Treatment: F_3,102.88_ = 6.728, *P* < 0.001; Condition: F_1,105.48_ = 0.069, *P* = 0.794; Treatment× Condition: F_3,103.12_ = 0.721, *P* = 0.542), and muscles (Fig. 3C; Treatment: F_3,86.44_= 5.668, *P* = 0.001; Condition: F_1,85.39_= 0.062, *P* = 0.804; Treatment× Condition: F_3,85.90_= 2.139, *P* = 0.101). Post-hoc pairwise comparisons revealed that the δ13C levels in the linoleic and oleic acids treatments in adult tissues in the experimental groups were significantly different from the control group only in the presence of brood (Fig. 3).

### Effect of FAs on diet consumption and colony fitness

**Fig. 4:**
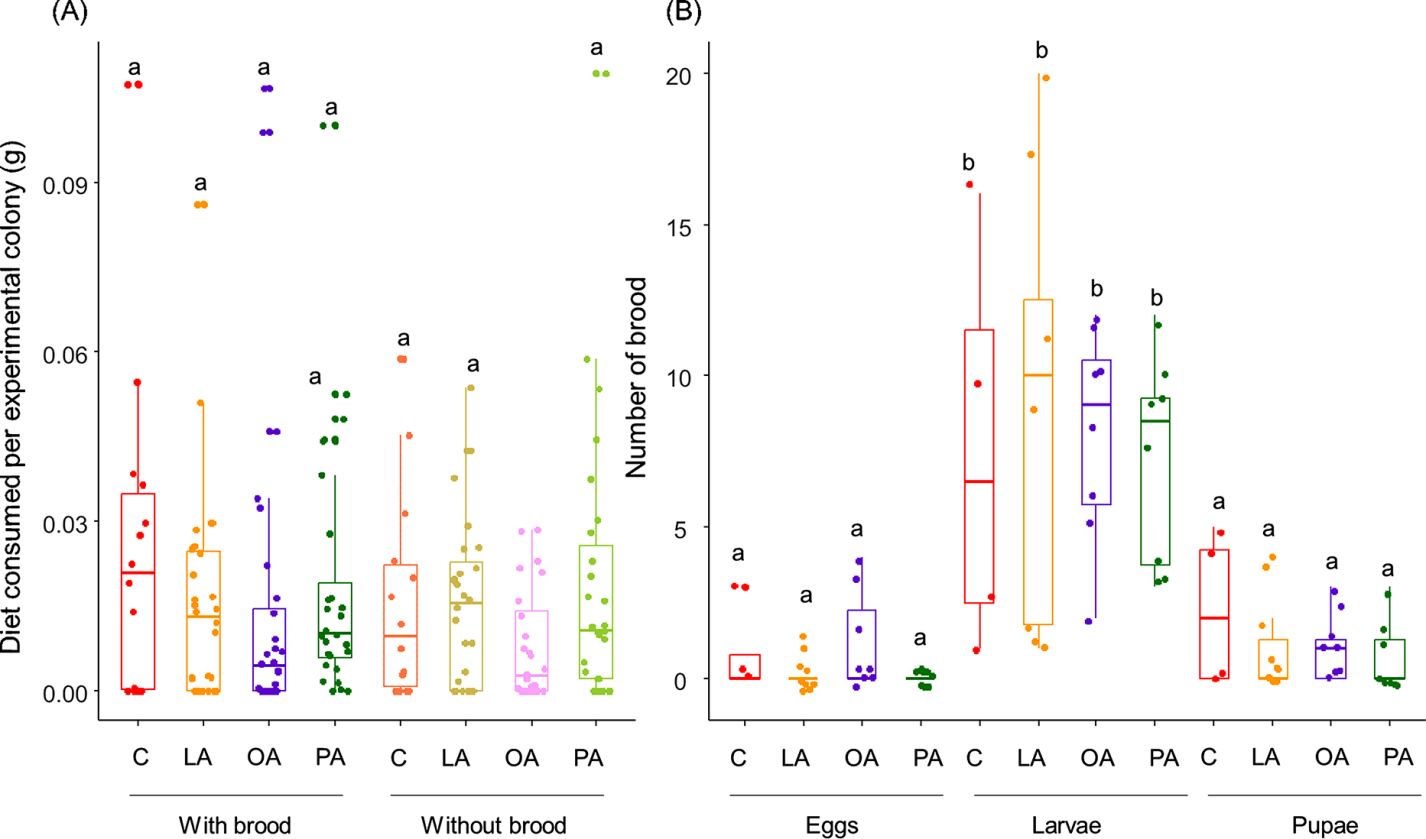
Effect of the FAs added in hornets’ diet on colonies’ consumption (**A**) and brood fitness (**B**). LA: linoleic acid, OA: oleic acid, PA: palmitic acid and C: control. The boxes represent the first and third quartiles and the median. The whiskers represent the maximum and minimum values. Groups that do not share the same letter are significantly different from each other (pairwise comparison with *P* < 0.05)

The quantity of meat consumed by the experimental colonies was not affected by the FAs added nor by the presence of brood (Fig. 4A; Treatment: F_3,187.00_ = 1.684, *P* = 0.172; Condition: F_1,187.00_= 1.521, *P* = 0.219; Treatment× Condition: F_3,187.00_= 0.616, *P* = 0.606). The FAs added in the hornet’s diet did not affect the number of larvae alive, pupae, or eggs laid by the workers (Fig. 4B; Treatment; F_3,69.00_= 1.162, *P* = 0.331; Type of brood: F_2,69.00_ =67.009, *P* < 0.001; Treatment× Type of brood: F_6,69.00_= 0.518, *P* = 0.792).

## Discussion

We found variations in oxidation and utilization of dietary FAs with different saturation levels, as well as with the life stage of the individual hornets. Larvae exhibited the ability to absorb and oxidize all tested FAs, with palmitic acid being oxidized at a notably higher rate. In contrast, adult hornets did not utilize any of the FAs tested as a metabolic fuel source. Surprisingly, larvae displayed reduced incorporation of linoleic acid into their bodily tissues while demonstrating an equal incorporation rate for oleic and palmitic acids. Pupal tissues, on the other hand, exhibited higher levels of oleic acid. In adults, both linoleic and oleic acids were found to be integrated into all examined body tissues (brain, fat body, and muscles), while palmitic acid incorporation into body tissues was minimal, corroborating findings from earlier studies (Dubiner et al. 2023; Seltzer et al. 2023). FAs diet enrichment did not affect the consumption and fitness of the experimental colonies.

In contrast to larvae, adult worker hornets did not utilize FAs as a source of metabolic energy. Yet, bumble bee workers readily oxidized linoleic and oleic acids (Seltzer et al. 2023). Insects possess a diverse array of strategies for modulating nutrient oxidation in accordance with their physiological state, including periods of nutrient scarcity or starvation (McCue et al. 2015). In the case of hornets, specific amino acid oxidation is influenced by their activity phase (Bodner et al. 2021). As such, it is plausible that the oxidation of FAs by adult hornets may be dependent on particular physiological or activity states.

Oleic acid is the predominant FA within the bodies of adult insects, including hornets (Falkenstein et al. 2001; Levin et al. 2013; Volov et al. 2021), and can be synthesized de novo in the body. Consequently, the presence of this FA across all life stages of hornets (larvae, pupae, and adult) is consistent with this established trend. Linoleic acid, however, in contrast to oleic and palmitic acids, is an essential FA that must be obtained from the diet (De Carvalho and Caramujo 2018). One could have expected this essential FA to be allocated within the body in higher concentrations, especially in larval and pupal tissues. In a previous study, we showed that the lipid composition of the hornet body is highly regulated and deficient in essential PUFAs, despite variations in climate and under artificially enriched PUFA diets (Volov et al. 2021). The current study corroborates these findings by demonstrating low levels of tissue incorporation of this essential FA. The most intriguing results of our experiments are the relatively high rate of oxidation and allocation of palmitic acid exhibited by the larvae, whereas adults, in our study and others, hardly utilize this FA. Palmitic acid, despite its crucial physiological functions, is usually negatively depicted for its detrimental health effects in humans (Carta et al. 2017). However, recent research has shown that the majority of ingested palmitic acid is not absorbed but rather excreted in feces (Dubiner et al. 2023). Given that hornet larvae do not defecate - and hence cannot egest palmitic acid- it is plausible that they have evolved mechanisms to enhance its absorption and utilization. A noteworthy consideration is the more intricate microbiome found in hornet larvae compared to adults (Cohen et al. 2023). This richer microbial community allow them to process more complex nutrients, and could potentially facilitate the absorption and utilization of SFA by breaking it into acetyl CoA. Further studies are necessary to better understand this phenomenon.

Larvae process complex nutrients in the food collected by adults and deliver it as a drop of liquid secretion containing carbohydrates and free amino acids, among other nutrients (Abe et al. 1991; Bodner et al. 2024; Hunt 2007; Ishay and Ikan 1968a). The difference in δ13C levels observed in the exhaled breath of adult hornets subjected to the two conditions – with or without brood –clearly illustrate this phenomenon. In the presence of brood, adult hornets, alongside the meat and sucrose solution, had access to the secretion produced by the larvae. Consequently, the composition of their diets became more diversified, including sugars synthesized by larvae from meat proteins via gluconeogenesis (Ishay and Ikan 1968a, b), leading to discernable differences in δ13C values. Moreover, our study seems to indicate that larvae could facilitate adult digestion of FAs, as the differences in adult tissues between control and isotopically labeled FAs groups were statistically significant only in presence of brood. However, it is important to note that although the difference between groups in the condition without brood did not yield statistically significant outcomes, the δ13C values detected in adult tissues fed with labeled linoleic and oleic acids were higher than those of the control group. This indicates that some individuals incorporated PUFAs and MUFAs into their body tissues even in the absence of larvae. In our study, adult hornets were randomly selected during nest collection. The workers’ age and/or task (i.e., nurse, forager, and guard) may influence their capacity to digest and utilize fatty acids within their body tissues. For instance, in honey bees, the behavioral task transition from nursing to foraging is characterized by the inability of workers to acquire dietary lipids (Toth et al. 2005). As task partitioning remains unknown in the Oriental hornet, further studies that consider the workers’ possible task and physiological age are necessary to understand the role of larvae in the digestion and allocation of fatty acids in adults.

We did not find any effect of the additional dietary FAs on the colony consumption or fitness. This is probably due to the very low concentration used to enrich our diet (0.2%). In bees (honey bees and bumbles bees) for example, only diets containing significantly elevated fat content affected consumption and fitness (Ruedenauer et al. 2020; Stabler et al. 2021).

In conclusion, contemporary research has significantly advanced our understanding of the physiological and health implications of different dietary fatty acids in both humans and animals. However, there remains a notable gap in knowledge regarding the absorption and oxidation dynamics of these different FAs. In this study, employing 13 C labeled fatty acids with different saturation levels, we elucidate key distinctions in the utilization of these FA by hornets. Our findings underscore the necessity of considering not only the degree of FA saturation but also the life stage of individuals when elucidating FA oxidation and allocation patterns.

## Data Availability

The raw data are available to download from Zenodo Repository, https://doi.org/10.5281/zenodo.13832513.
